# A Novel Active Targeting Preparation, Vinorelbine Tartrate (VLBT) Encapsulated by Folate-Conjugated Bovine Serum Albumin (BSA) Nanoparticles: Preparation, Characterization and *in Vitro* Release Study

**DOI:** 10.3390/ma5112403

**Published:** 2012-11-20

**Authors:** Yong Li, Xiuhua Zhao, Yuangang Zu, Xue Han, Yunlong Ge, Weiguo Wang, Xinyang Yu

**Affiliations:** Key Laboratory of Forest Plant Ecology, Northeast Forestry University, Harbin 150040, China; E-Mails: liyong19870828@sina.cn (Y.L.); xiuhuazhao@nefu.edu.cn (X.Z.); ameko19871029@126.com (X.H.); wo-geyunlong@163.com (Y.G.); wang901234@163.com (W.W.); yuxinyangsarah@163.com (X.Y.)

**Keywords:** vinorelbine tartrate, bovine serum albumin, folate, nanoparticles, target drug delivery

## Abstract

Vinorelbine tartrate (VLBT), as a kind of high hydrophilic and temperature-induced degradation drug, was prepared into nanoparticles by a desolvation procedure. Bovine serum albumin (BSA), as a drug carrier, was stabilized by chemical cross-linking with glutaraldehyde. Firstly, the optimization process of preparing VLBT-loaded BSA nanoparticles (VLBT-BSANPs) was accomplished using response surface methodology (RSM) by desolvation. Then VLBT-BSANPs were conjugated with folate, namely Fa-BSANPs-VLBT. Hence targeting drug carrier delivery system loading VLBT was produced. In this study, the characteristics of the nanoparticles, such as the amount of folate conjugation, surface morphology, surface chemistry, physical status of VLBT in Fa-BSANPs-VLBT, stability of Fa-BSANPs-VLBT with mannitol and *in vitro* drug release behavior were all investigated. The VLBT-BSANPs were obtained under optimum conditions, with a mean particle size (MPS) of 155.4 nm and a zeta potential (ZP) of −32.97 mV at a pH value of 5.4. Drug loading efficiency (DLE) and drug entrapment efficiency (DEE) of this obtained drug were approximately 45.6% and 90.6%, respectively.

## 1. Introduction

**Figure 1:**
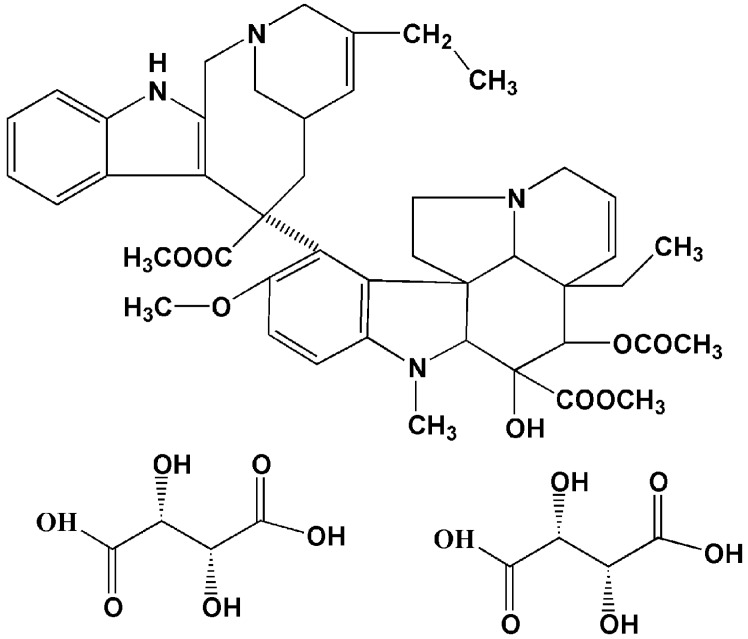
Chemical structure of vinorelbine tartrate (VLBT).

Vincristine, vinblastine and vinorelbine (VLB), three kinds of vinca alkaloids, represent one of the most widely used classes of antineoplastic agents [1]. Their cytotoxicity against cancer is based on their ability to inhibit the dynamics and assembly behavior of microtubule during the metaphase, and cell growth is arrested during metaphase [2,3]. VLB (5'-nor-anhydrovinblastine), a semisynthetic vinca alkaloid that differs from other vincas by a modification of the catharanthine moiety, exhibits a broader spectrum of anti-tumor activity and reduced neurotoxicity compared to the naturally occurring vincas [4]. The highest activity of this drug has been proven in patients with non-small-cell lung cancer (NSCLC) [5,6,7] and advanced breast cancer (ABC) [8,9,10]. It is currently registered for the treatment of ABC and NSCLC in most countries [11]. An injectable formulation of VLB (Navelbine^®^ IV), which mainly consists of vinorelbine tartrate (VLBT) (Figure 1) developed by Pierre Fabre Medicament France, is now widely marketed for the treatment of two kinds of diseases in many countries [12,13,14,15,16]. However, short-duration and non-cumulative granulocytopenia are the major dose-limiting toxicities, and other side effects, such as nausea and vomiting, constipation, peripheral neuropathy and reversible alopecia, can occur as well, but are generally mild [17]. Moreover, Navelbine® IV is not an optimal drug delivery system because of its vesicant action. It is very well known that this action tends to cause venous irritation and phlebitis when Navelbine^®^ IV is directly administered intravenously as an aqueous solution. Venous irritation is reported as injection site reactions, local reactions, and superficial phlebitis, whereby the symptoms include erythema, pain at the injection site, vein discoloration and tenderness along the vein [17]. Thus, it is necessary to find a new strategy to reduce the venous irritation produced by aqueous injections of VLB. A stable oral dosage form of VLB (Navelbine^®^ Oral), a soft gelatin capsule filled with VLB ditartrate/ethanol/water/glycerol/macrogol 400 solution, which was developed in 1994 by Pierre Fabre Medicament, is also available on the market [18]. The advantages of the oral formulation chemotherapy development are patient pharmaco-economic issues, patient preference and cost savings compared to i.v. administration [19,20]. However, the severity of the side effects caused by the oral administration caused was greater than with the i.v. form.

In view of the disadvantages of both Navelbine^®^ IV and Navelbine^®^ Oral, the present study was carried out to discover an ideal carrier or a novel strategy for VLB so as to reduce the severe venous irritation, enhance the anti-tumor activity and improve patient tolerance. It has been reported that many kinds of drug delivery systems loading VLB have been developed. For example, much attention has been focused on the study of liposome-encapsulated VLB [2,21,22,23] and solid lipid nanoparticles loading VLB bitartrate [24,25]. However, no study has been established using vinorelbine tartrate loaded folate-conjugated bovine serum albumin nanoparticles (Fa-BSANPs-VLBT), which are also considered as a formulation belonging to drug delivery systems. In this study, we have tried to prolong circulation and improve biodistribution for VLBT by using folate-conjugated bovine serum albumin (BSA) nanoparticles (Fa-BSANPs). Fa-BSANPs, used as a targeting drug carrier system, are aimed at the specific targeting of tumor cells or tumor tissues to enhance the efficacy and reduce the toxicity of compounds with anti-tumor activity. 

Before our study, Zu and colleagues prepared vinblastine sulfate (VBLS)-loaded folate conjugated bovine serum albumin (BSA) nanoparticles [26]. In this VBLS study, nanoparticles loaded with VBLS were prepared through two steps, which led to great material and time loss. The resulting Fa-BSANPs showed a drug entrapment efficiency (DEE) of 84.83% and a drug loading efficiency (DLE) of 42.37%, respectively. To get rid of the drawbacks in the previous study, we tried to use a one-step method to prepare VLBT encapsulated by BSA nanoparticles. The results indicated that DEE and DLE of the obtained drug were approximately up to 90.6% and 45.6%, respectively. Comparatively speaking, the one-step method shows its advantages in improving DLE and DEE to some degree. In other words, the one-step method shows a bright application prospect in the process of preparing targeting drug delivery systems. In this paper, a nanoencapsulation procedure of desolvation and subsequent cross-linkage were used to prepare VLBT-BSANPs. The process of preparing VLBT-BSANPs was optimized through response surface methodology (RSM). In the present study, the surfaces of VLBT-BSANPs were decorated by covalent attachment of folate to enable the tumor cell-targeting ability. Characterizations of the nanoparticles, including DEE, DLE, mean particle size (MPS) and zeta potential (ZP), were carried out. In addition, the amount of folate conjugation, surface morphology, physical status of VLBT in Fa-BSANPs-VLBT and surface chemistry were also investigated. Having prepared Fa-BSANPs-VLBT, we added mannitol as a lyoprotectant to prevent Fa-BSANPs-VBLST from aggregation for a freeze-drying process in the purpose of long-term stability and preservation of the original pharmaceutical and biological properties of the products. The release properties and stability of the dry power were tested in the present work. 

## 2. Results and Discussion

### 2.1. Optimization Study

When the nanoparticles are injected *in vivo*, their particle size plays a key role in determining body distribution and promoting their access into cancer cells after the folate has targeted cancer tissues. A statistical approach using a central composite design was used to optimize particle size and maximize DLE. The four-factor and five-level central composite design (CCD) matrix and experimental results obtained are presented in Table 1. The analysis results of the effect of selected process parameters indicated that not all of the main effects were significant using RSM. Therefore, a polynomial regression modeling was performed between the response variable (DLE % and MPS nm) and the corresponding coded values (*X*_1_, *X*_2_ and *X*_4_), and finally, the best-fitted model equations are shown as;


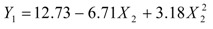
(1)


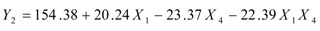
(2)

**Figure 2:**
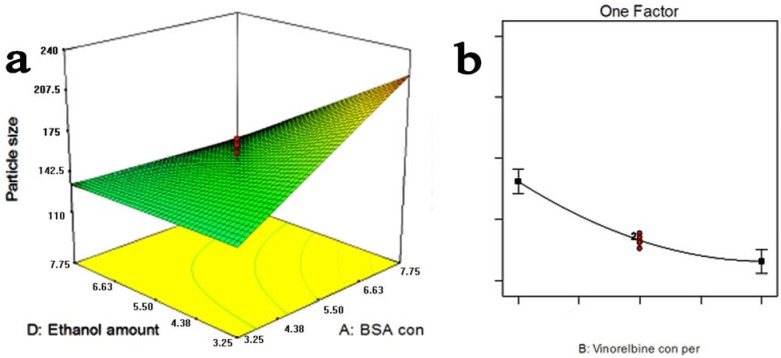
(**a**) Response surface plots for mean particle size of the vinorelbine tartrate loaded bovine serum albumin nanoparticles (VLBT-BSANPs) showing interaction between bovine serum albumin (BSA) concentration and the ratio of ethanol and VLBT solution (v/v); (**b**) Response surface plots for drug loading efficiency (DLE) of the VLBT-BSANPs.

According to the ANVOA, *X*_1_ and *X*_4_ were found to have significant effects on the MPS. So these variables were chosen to plot the response surface for the MPS while holding *X*_2_ and *X*_3_ at central points, 0.55 mg/mL and 3.25 mL/min, respectively. As can be seen from Figure 2a, MPS increased distinctly as the BSA concentration increased. MPS had a minor increase as the BSA concentration was increased up to 5.5 mg/mL, at this rate, MPS had a slight increase when the BSA concentration (*X*_1_) was increased. According to the ANVOA, the ratio of BSA concentration and VLBT concentration (*X*_2_) had pronounced effects on DLE. As shown in Figure 2b, the BSA concentration, ethanol rate and the ratio of the ethanol amount and VLBT solution, (namely 5.5 mg/mL and 3.25 mL/min) and DLE decreased with the increase of the ratio of BSA and VLBT (c/c, *X*_2_).

**Table 1:** The four-factor central composite design matrix and performance values.

Run	Variables				Responses	
	*X*_1_	*X*_2_	*X*_3_	*X*_4_	*Y*_1_ (%)	*Y*_2_ (nm)
1	5.50	5.00	3.25	5.50	13.14	151.1
2	7.75	3.00	4.63	7.75	22.47	114.6
3	7.75	7.00	4.63	3.25	11.25	219.5
4	3.25	3.00	1.88	3.25	21.73	167.7
5	3.25	7.00	1.88	7.75	8.72	79.7
6	5.50	5.00	3.25	5.50	12.32	159.0
7	3.25	3.00	4.63	7.75	25.43	119.7
8	1.00	5.00	3.25	5.50	12.22	111.7
9	5.50	9.00	3.25	5.50	9.26	142.6
10	5.50	5.00	3.25	1.00	31.64	230.4
11	7.75	3.00	1.88	3.25	19.37	246.3
12	5.50	5.00	3.25	5.50	11.35	164.7
13	7.75	7.00	1.88	3.25	10.61	176.8
14	3.25	7.00	4.63	3.25	8.91	158.6
15	3.25	7.00	4.63	7.75	6.03	142.1
16	3.25	3.00	1.88	7.75	17.55	147.3
17	5.50	1.00	3.25	5.50	46.91	171.8
18	3.25	3.00	4.63	3.25	21.60	54.2
19	3.25	7.00	1.88	3.25	9.94	141.4
20	5.50	5.00	0.50	5.50	11.05	210.0
21	5.50	5.00	3.25	5.50	13.83	170.0
22	5.50	5.00	3.25	5.50	13.10	149.3
23	10.00	5.00	3.25	5.50	14.27	192.4
24	7.75	7.00	4.63	7.75	9.96	115.7
25	5.50	5.00	3.25	10.00	10.93	162.2
26	5.50	5.00	3.25	5.50	12.60	167.0
27	7.75	7.00	1.88	7.75	8.57	185.7
28	5.50	5.00	6.00	5.50	12.09	103.5
29	7.75	3.00	4.63	3.25	9.27	220.6
30	7.75	3.00	1.88	7.75	22.18	55.9

Nanoparticles with the MPS of 100 to 200 nm had an enhanced permeability and retention (EPR) effect, which allowed the preferred accumulation of drug-loaded nanoparticles within tumors [27]. Moreover, pegylated liposomes with an MPS of 120 to 160 nm were found to circulate in the blood for long periods of time [28]. In view of the two facts, 150 nm was chosen as the target for the optimization of the MPS of the VLBT-BSANPs, while maximization was the chosen aim for the optimization of DLE. The optimum values of the variables were obtained by graphical and numerical analyses using the Design-Expert^®^ software and based on the criterion of desirability. The optimized formulation was achieved with 3.29 mg/mL BSA, 3mg/mL VLBT, 2.84 mL/min of ethanol rate, and 3.44 fold of the ethanol amount (ethanol amount: VLBT solution, 3.44:1, v:v). The VLBT-BSANPs were obtained under optimum conditions, with a MPS of 155.4 nm and a ZP of −32.97 mV. The DLE and DEE of this obtained drug were approximately up to 45.6% and 90.6%, respectively. Thereafter, all of the following experiments were conducted using VLBT-BSANPs produced by this optimized formulation.

### 2.2. Characterization of FA-BSANPs-VLBT

#### 2.2.1. Folate Content Associated with the VLBT-BSANPs

Due to the low accuracy of UV spectrophotometry, high performance liquid chromatography (HPLC) was used to achieve a quantitative test on the folate content associated with the VLBT-BSANPs. The result indicated that 5.67 μg *N*-hydroxysuccinimide ester of folate (NHS-folate) was conjugated with 1 mg BSANPs according to the corresponding standard curve, namely *y* = 2877*x* + 645.9, *R*^2^ = 0.9995 (*y*: NHS-folate concentration, μg/mL; *x*: peak area, AU*min). In Karsten Ulbricha^’^s research, folate-conjugated human serum albumin (HSA) nanoparticles showed favorable targeting efficiency for tumor cells when 7.40 ± 0.90 μg folate was bound per mg HSANPs [29]. Moreover, when folate content was determined to be 0.38 and 6.42 molecules folate per molecule HSA, which meant that 2.52 to 42.570 μg folate was bound per mg human serum albumin nanoparticles (HSANPs), folic acid-functionalized human serum albumin nanocapsules also had favorable targeting efficiency for chronically activated macrophages[30].

#### 2.2.2. Surface Morphology of Nanoparticles

**Figure 3:**
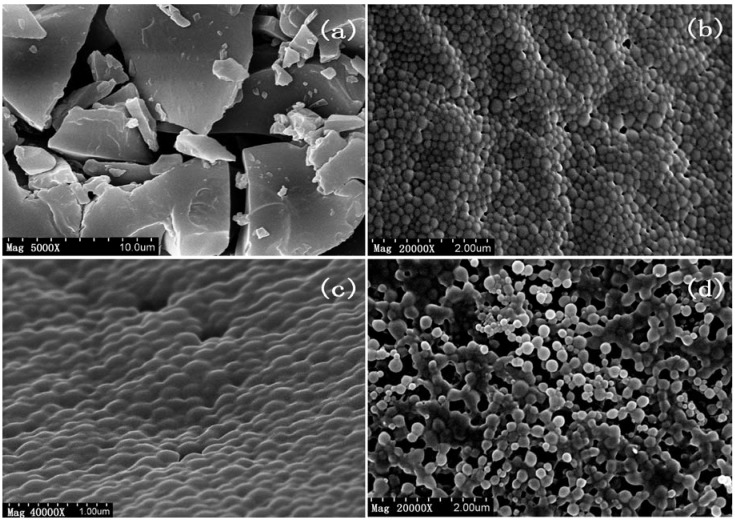
Scanning electron microscopy (SEM) image of raw VLBT and VLBT-loaded nanoparticles. (**a**) Raw VLBT; (**b**) VLBT-BSANPs; (**c**) Fa-BSANPs-VLBT; (**d**) Fa-BSANPs-VLBT with mannitol.

In order to examine and compare the surface characteristics of the nanoparticles resulting from each step in preparing the optimal formulation of Fa-BSANPs-VLBT, they were prepared and scanned using scanning electron microscopy (SEM). In Figure 3a, particles were in irregular bulk form, with diameters in the 2–10 μm range. In contrast, VLBT-loaded nanoparticles (Figure 3b–d) were seen as roughly spherical nanoparticles with a uniform distribution. 

The VLBT-BSANPs (Figure 3b) were evenly distributed in particle size and had an average size of 200 nm, which was in accordance with the MPS of VLBT-BSANPs obtained under optimum conditions measured by DLS, namely 155.4 nm. However, the conjugation of the folate with the BSANPs changed the surface characteristics of VLBT-BSANPs, and they aggregated each other (Figure 3c). The SEM images of nanoparticles freeze-dried with mannitol (Figure 3d) revealed that the aggregation still existed. The particle size of Fa-BSANPs-VLBT, which had a roughly spherical surface morphology, namely about from 150 to 200 nm, as seen in Figure 3d, was in favor of circulation in blood for long periods of time and of sustaining the release property of this drug delivery system [27,28].

The aggregation phenomenon directly caused that the MPS of Fa-BSANPs-VLBT with mannitol increased to about 300 nm (Table 2) measured by DLS, which was bigger than the particle size in Figure 3d. However, in favor of mannitol, Fa-BSANPs-VLBT was dispersed very well. Moreover, no drug crystals were visible in Fa-BSANPs-VLBT, and Fa-BSANPs-VLBT with mannitol demonstrated the stability and applicability of the obtained drug delivery system.

#### 2.2.3. Surface Chemistry

**Figure 4:**
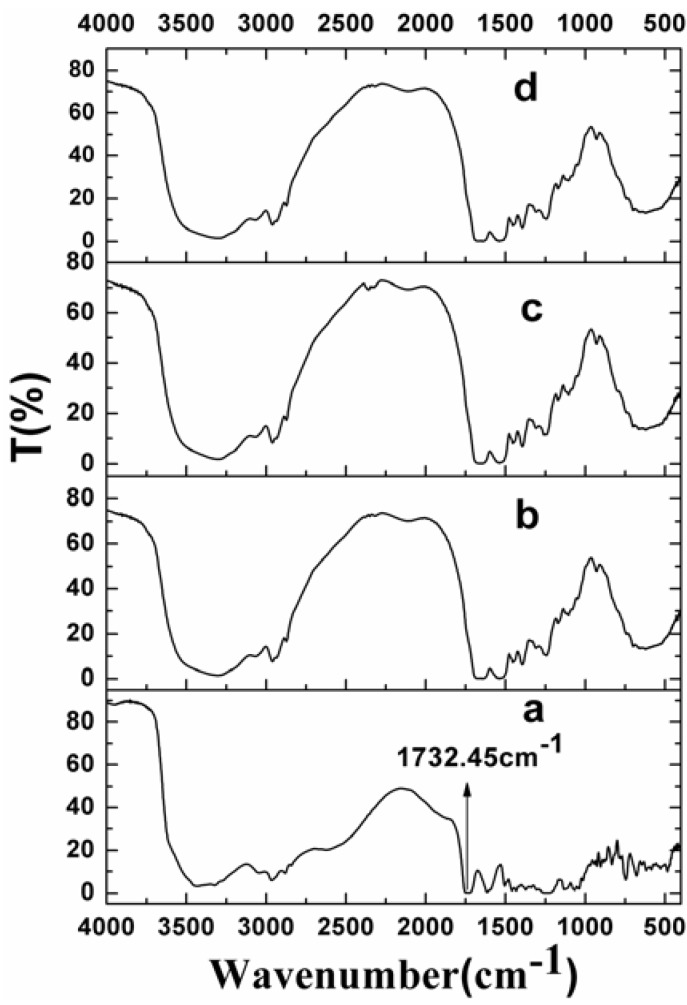
Fourier transform infrared (FTIR) spectra of raw VLBT and VLBT-loaded nanoparticles. (**a**) Raw VLBT; (**b**) VLBT-BSANPs; (**c**) Fa-BSANPs-VLBT; (**d**) Fa-BSANPs-VLBT with mannitol.

Figure 4 shows the fourier transform infrared (FTIR) spectroscopy of raw VLBT and VLBT-loaded nanoparticles. In this Figure, only raw VLBT presented a remarkable absorption peak (a very low transmission) at 1732.45 cm^−1^ due to the COOH bond, which proved that VLBT had been entirely wrapped up inside the BSA. The phenomenon that VLBT was entirely wrapped up inside the BSA made VLBT circulate in blood for long periods of time and improved the stability of VLBT in circulation.

#### 2.2.4. Physical Status of VLBT in Fa-BSANPs-VLBT

**Figure 5:**
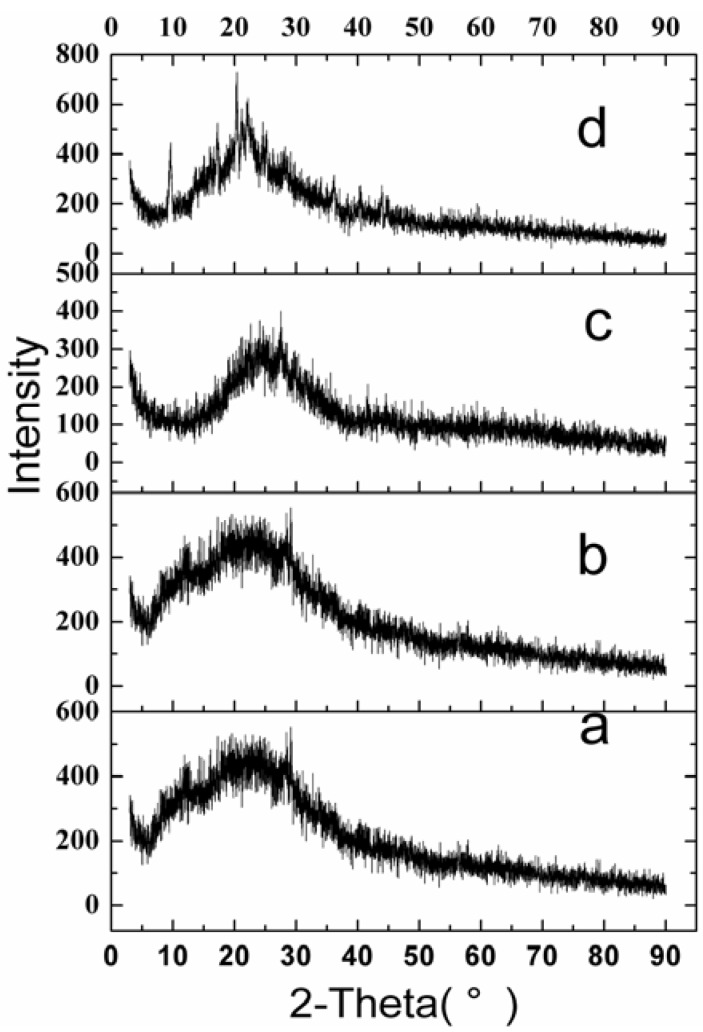
X-ray diffraction (XRD) patterns of raw VLBT and VLBT-loaded nanoparticles. (**a**) Raw VLBT; (**b**) VLBT-BSANPs; (**c**) Fa-BSANPs-VLBT ; (**d**) Fa-BSANPs-VLBT with mannitol.

The X-ray diffraction (XRD) results for raw VLBT, VLBT-BSANPs, Fa-BSANPs-VLBT and Fa-BSANPs-VLBT with mannitol are shown in Figure 5. Raw VLBT, VLBT-BSANPs and Fa-BSANPs-VLBT did not show any peak from 3° to 90°. Nevertheless, the peaks of Fa-BSANPs-VLBT with mannitol at 9.4°, 17.2° and 20.2°, showed the crystalline structure of mannitol. That raw VLBT did not present any peak indicating that VLBT might be present in an amorphous state. 

**Figure 6:**
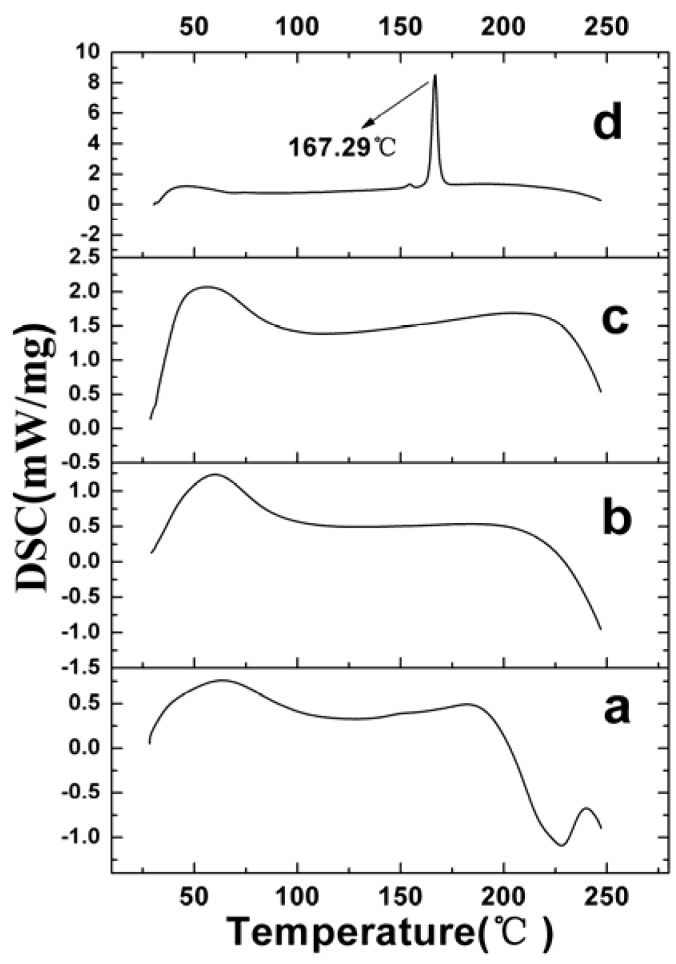
Differential scanning calorimetry (DSC) patterns of raw VLBT and VLBT-loaded nanoparticles. (**a**) Raw VLBT; (**b**) VLBT-BSANPs; (**c**) Fa-BSANPs-VLBT ; (**d**) Fa-BSANPs-VLBT with mannitol.

Figure 6 shows the results of differential scanning calorimetry (DSC). The raw VLBT did not exhibit an obvious melting process, which implied the noncrystalline form of VLBT. No melting process was observed for VLBT-BSANPs and Fa-BSANPs-VLBT, which showed that both were nanostructured and noncrystalline. However, Fa-BSANPs-VLBT with mannitol exhibited an obvious melting process, with a peak of 167.29 °C, which implied the crystalline form of mannitol. The results were consistent with the results of the XRD analysis.

**Figure 7:**
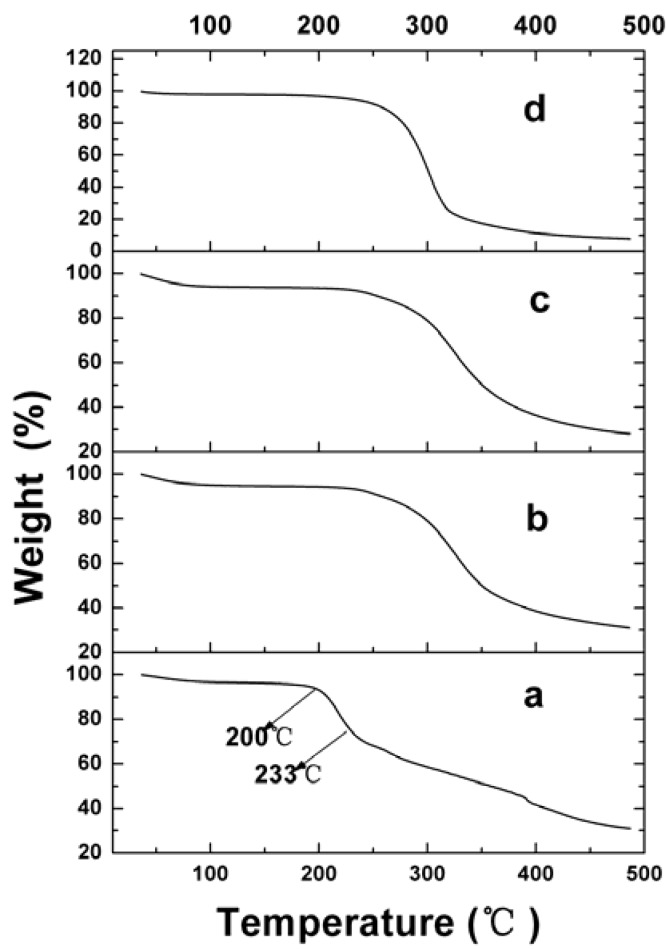
Thermogravimetric analyzer (TGA) results of raw VLBT and VLBT-loaded nanoparticles. (**a**) Raw VLBT; (**b**) VLBT-BSANPs; (**c**) Fa-BSANPs-VLBT; (**d**) Fa-BSANPs-VLBT with mannitol.

The thermogravimetric analyzer (TGA) results, used to examine the thermal property of raw and VLBT-loaded nanoparticles, are shown in Figure 7. Raw VLBT was observed to moderately lose weight from 36 to 180 °C, due to its water loss. Then its weight decreased quickly from 200 to 233 °C, and the rate of weight loss slowed down in an approximate linear model due to its strong vaporization, followed by its decomposition. Meanwhile, the VLBT-loaded nanoparticles began to lose weight quickly from about 250 °C.

Moreover, the TGA result of VLBT-loaded nanoparticles indicated that they had a higher weight loss percentage compared with the raw VLBT at the same temperature. This was possibly due to the fact that VLBT-loaded nanoparticles with small MPS had a higher specific surface area, which was in favor of cellular uptake of cancer cells, and hence had a higher specific surface energy, which subsequently lead to an easier vaporization and earlier decomposition energy.

### 2.3. *In Vitro* VLBT Release from Fa-BSANPs-VLBT

**Figure 8:**
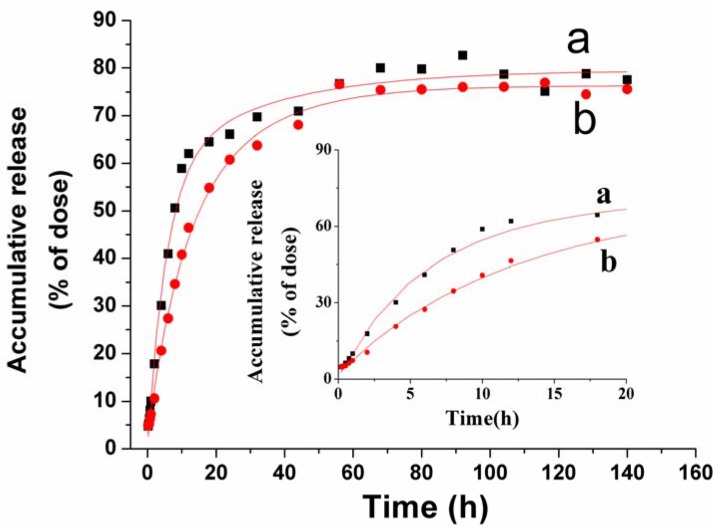
The release profiles of raw VLBT and Fa-BSANPs-VLBT with mannitol. (**a**) Raw VLBT; (**b**) Fa-BSANPs-VLBT with mannitol.

The Fa-BSANPs-VLBT powder with mannitol was reconstituted to carry out *in vitro* drug release experiments over 140 h, the results of which are shown in Figure 8. An initial burst of more than 60% of the raw VLBT in the first 12 h could be observed. And then a slow release happened up to 56 h and a cumulative release reached 76%. The drug carrier system had a burst release at 20 h and a slow release up to 56 h, releasing approximately 60% and 76% of VLBT, respectively (Figure 7b). The release rate of the raw VLBT was distinctively higher than Fa-BSANPs-VLBT in the first 24 h, which played a key role in determining the therapeutic efficacy. It is the evident sustain release in the first 24 h that allows Fa-BSANPs-VLBT to have a better therapeutic efficacy than raw VLBT. Moreover, the observed release curves were analyzed using the Originpro (Version 8.0) software (OriginLab Corporation, Northampton, USA). The release characteristic of raw VLBT was in accordance with the Higuchi equation of *y* = −58.35*e*^(−*x*/5.56)^ − 20.47*e*^(−*x*/33.01)^ + 79.55 (*R*^2^ = 0.9934). Meanwhile, the release of Fa-BSANPs-VLBT with mannitol corresponded with the Higuchi equation of *y* = −33.36*e*^(−*x*/8.86)^ − 40.59*e*^(−*x*/21.76)^ + 76.32 (*R*^2^ = 0.9967). The observed release curve indicated that the drug carrier system had a sustained release property that facilitated the application of nanoparticles for the delivery of anticancer drugs.

### 2.4. Stability of Fa-BSANPs-VLBT

The MPS of Fa-BSANPs-VLBT measured by DLS was bigger than that observed by SEM owing to aggregation. Moreover, the conjugation of the folate with the BSANPs and lyophilization of Fa-BSANPs-VLBT increased the MPS of VLBT-BSANPs from155.4 nm to about 300 nm, and the ZP of VLBT-BSANPs changed from −32 mV to about −17 mV (Table 2) when the pH value was 5.3. The short-term stability study showed that Fa-BSANPs-VLBT was physically and chemically stable for up to 8 h at room temperature (Table 2). There was no significant change in MPS and ZP. The precept of the stability study originated from the evaluation of Abraxane from FDA (US Food and Drug Administration). The high stability of Fa-BSANPs-VLBT for MPS and ZP in 8 h was in great favor of storage and stable therapeutic efficacy.

**Table 2:** Stability of Fa-BSANPs-VLBT.

Time (h)	0	1	2	4	8
MPS (nm)	297.4	326.3	330.7	341.5	364.5
ZP (mV)	−17.37	−15.59	−17.50	−17.30	−16.26

## 3. Experimental Section

### 3.1. Materials

Folate, bovine serum albumin, trypsin, phosphate-buffered saline (pH 7.4), BSA trypsin and 2,4,6-trinitrobenzenesulfonic acid (TNBS) were all obtained from Sigma Aldrich (St. Louis, MO, USA). The VLBT was kindly provided by Hisun Pharmaceutical Co. Ltd. (Zhejiang, China). Acetonitrile and ethanol were of high performance liquid chromatography grade. *N*-hydroxysuccinimide (NHS, purity > 99.0%), 1,3-dicyclohexyl-carbodiimide (DCC, purity > 99.0%), Dimethyl Sulphoxide (DMSO, purity > 99.5%), ethanol (purity > 99.5%) and the other reagents were all of analytical grade.

### 3.2. Preparation of Fa-BSANPs-VLBT 

#### 3.2.1. Preparation of the *N*-Hydroxysuccinimide Ester of Folate (NHS-folate)

Three grams of folate were dissolved in 60 mL dimethyl sulfoxide (DMSO) containing 1.5 mL triethylamine, and then 2.82 g DCC and 1.56 g NHS were added to the solution under stirring. The mixture was stirred for 12 h at room temperature. The insoluble dicyclohexyl urea was removed by filtration, and then the filtrate was poured into ice-cold anhydrous ether solution containing 30% acetone, followed by centrifugation at 3000 g for 5 min at 4 °C and washed twice using ether. Finally, NHS-folate, a delicate light yellow solid powder, was obtained by drying at room temperature [31].

#### 3.2.2. Preparation and Optimization of VLBT-Loaded BSANPs by RSM

The VLBT-BSANPs were prepared as previously described using a desolvation procedure. In the preliminary trials, we found that if VLBT was dissolved in purified water, the formation of BSANPs as drug carriers was unstable with precipitation. Thus the pH for the VLBT water solution was adjusted to about six with a NaHCO_3_ buffer. The VLBT was loaded mainly through electrostatic interaction between positive charges of the drug and negative charges of the BSANPs, when the pH value of the solution was higher than the isoelectric point of BSA. Huang and colleagues have reported this mechanism of loading drugs [32]. 

**Figure 9:**
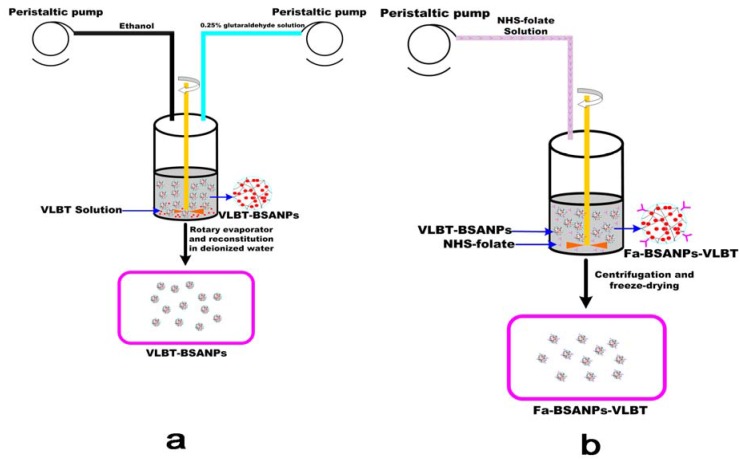
Schematic description of the preparation procedure of Fa-BSANPs-VLBT: (**a**) VLBT-BSANPs were produced with a desolvation technique; (**b**) VLBT-BSANPs powder was reconstituted in deionized water, followed by the addition of the NHS-folate solution with a peristaltic pump, NHS-folate was first dissolved under alkaline conditions.

As illustrated in Figure 9a, after VLBT was first dissolved in NaHCO_3_, the buffer solution together with BSA, and subsequently the desolvation agent of ethanol were added dropwise into the aqueous BSA and VLBT solution under magnetic stirring with a peristaltic pump TI/62/20 (Medorex, Norten-Hardenberg, Germany) at room temperature. The opalescent suspension was formed spontaneously and was further examined as nanoparticles. After the desolvation process, 0.25% glutaraldehyde in water (v/v) solution was added to crosslink the amino groups of the VLBT-BSANPs. Crosslinking was initiated under stirring of the colloidal suspension over a time period of 24 h. Ethanol in the prepared sample was eliminated by evaporation using a rotary evaporator R201BL (SENCO, Shanghai, China) at 40 °C, and then the VLBT-BSANPs were redispersed to the original volume with a NaHCO_3_ buffer solution. The redispersion process was performed in an ultrasonication bath TI-H-5 (Elma, Singen, Germany) for about 3 min. This redispersion solution was centrifuged (20,000 ×g, 10 min) to separate the free VLBT. After each centrifugation, the residue was redispersed to the original volume, with deionized water, using a mechanical stirrer. This purification process was repeated three times. Finally, the supernatant from each centrifugation was collected to determine the DEE and DLE. The nanoparticles settled were obtained correspondingly before lyophilization.

The above-described VLBT-BSANPs preparation procedure was optimized by a four-factor, five-level central composite design (CCD), with BSA concentration (mg/mL, *X*_1_), the ratio of BSA concentration and VLBT concentration (BSA:VLBT, c/c, *X*_2_), the rate of adding ethanol (ethanol rate, mL/min, *X_3_*), and the ratio of ethanol and VLBT solution (v/v, *X*_4_) as the independent variables (Table 3). The levels of those independent variables were based on preliminary trials. DLE (*Y*_1_) and MPS (*Y*_2_) of the resulting VLBT-BSANPs were used as response variables. Design-Expert^®^ Version 7.0.0 software (State-Ease Inc., Minneapolis, MN, USA) was applied to generate and evaluate the statistical experimental design.

**Table 3:** Factors and their levels in response surface methodology **(**RSM) designs.

Independent variables	Symbol	Coded levels		
		−1	0	1
BSA concentration(mg/mL)	*X*_1_	3.25	5.5	7.75
BSA:VLBT (c/c)	*X*_2_	3	5	7
ethanol rate(mL/min)	*X*_3_	1.88	3.25	4.63
Ethanol:VLBT solution (v/v)	*X*_4_	3.25	5.5	7.75

#### 3.2.3. Preparation of Fa-BSANPs-VLBT

As shown in Figure 9b, a certain amount of NHS-folate was dissolved in 1 mL NaHCO_3_ buffer solution (0.2 M, pH = 10) and added slowly to the VLBT-BSANPs solution while stirring. The reaction was maintained for 120 min at room temperature. Then the mixture was centrifuged at 10,000 rpm for 20 min three times and washed twice with NaHCO_3_ buffer solution, followed by freeze-drying. 

#### 3.2.4. Lyophilization of Fa-BSANPs-VLBT

The lyophilization of Fa-BSANPs-VLBT prepared in deionized water was performed using a Gamma 2-20 apparatus (Christ, Germany) to make sure the physical stability of the ultimate product along with the addition of mannitol as lyoprotectant (Fa-BSANPs-VLBT:mannitol, 1:1, w/w). The prepared Fa-BSANPs-VLBT was redispersed in 4 mL deionized water in 8 mL glass vials after purification and centrifugation. The mixture was prefrozen in the refrigerator (−20 °C) for 12 h and subsequently lyophilized at −40 °C for 24 h, followed by a secondary drying phase of 12 h at 20 °C.

### 3.3. Characterization of the Fa-BSANPs-VLBT

#### 3.3.1. Determination of Folate Content Associated with the VLBT-BSANPs

The amount of NHS-folate conjugated with VLBT-BSANPs was determined by high performance liquid chromatography (HPLC) using a Diamonsil C_18_ column, (250 mm × 4.6 mm, 5 μm; Dikma Technologies, Beijing, China) at ambient temperature. The mobile phase consisted of 50 mM phosphate buffer solution containing 8% methanol, which was adjusted to pH 6.3 with concentrated KOH solution. The system was run isocratically at a flow rate of 1 mL/min, and NHS-folate was detected at 254 nm. Briefly, the Fa-BSANPs-VLBT was digested by trypsin (50 g/mg BSA). Then the resultant tryptic hydrolysis of the Fa-BSANPs-VLBT was examined by HPLC. 

#### 3.3.2. Drug Loading and Encapsulation Efficiency 

The free VLBT in the supernatant was examined by HPLC. The total VLBT was the feed VLBT during preparation process of Fa-BSANPs-VLBT. In Formula 4, Fa-BSANPs-VLBT referred to the lyophilized powder of Fa-BSANPs-VLBT. A Waters HPLC (Waters Corporation, Milford, MA, USA), consisting of a Waters 600 Controller equipped with a Waters 717 plus autosampler, and a Waters 2487 UV detector, were used. The samples were chromatographed at 25 °C by injecting 10 μL sample into a Diamonsil C_18_ column. The mobile phase was a mixture of acetonitrile and 50 mM phosphate buffer solution containing 1% triethylamine (60/40, v/v; the pH of aqueous phase was adjusted to 4.0 with H_3_PO_4_), running at a flow rate of 1 mL/min. Detection was accomplished at 268 nm. DEE and DLE were calculated using equations as previously reported by Park and colleagues [33].

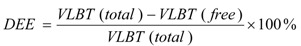
(3)

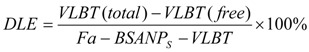
(4)

#### 3.3.3. MPS and ZP 

The obtained nanoparticles were analyzed for their MPS and ZP by dynamic light scattering (DLS) equipment (ZetaPALS, Brookhaven Instruments) with a He-Ne laser (632.8 nm, 35 mW) as light source. The measurement was performed in triplicate at 25 °C and with angle detection at 90° in 4 mm diameter cells with a field strength of 10 V/cm. The samples were prepared by diluting the nanoparticle suspension with deionized water. The data were obtained from the average of several measurements.

#### 3.3.4. Surface Morphology of Nanoparticles 

The morphology of the samples was determined by using scanning electron microscope (SEM, S4800, Hitachi). The total number of particles scanned was not less than 100. Samples were coated by gold before examination (cathode dispersion).

#### 3.3.5. Surface Chemistry

The samples were diluted with KBr mixing powder at 1% and pressed to obtain self-supporting disks, separately. The Fourier transform infrared (FTIR) spectrum was obtained by IRAffinity-1 (SHIMADZU, Japan) and recorded in the wave number range of 4000–500 cm^−1^ at a resolution of 4 cm^−1^.

#### 3.3.6. Physical Status of VLBT in Fa-BSANPs-VLBT

The X-ray diffraction (XRD) patterns were collected in transmission using an X-ray diffractometer with a rotating anode (Philips, Xpert-Pro, The Netherlands). The scanning rate was 5°/min, and the diffraction angle (2θ) was recorded at 3°–90° .The samples were irradiated using a Cu target tube at 30 mA and 40 kV. 

Thermal analysis was carried out using differential scanning calorimetry (DSC, TA instruments, model DSC 204) for the samples prepared in this experiment. All thermal analyses were performed in an inert atmosphere (N_2_). Analysis was performed for 5.0 mg samples at a temperature heating rate of 10 °C/min and a temperature range of 30–225°C.

The thermal stability of samples was also tested with a thermogravimetric analyzer (TGA, Diamond TG/DTA Perkin-Elmer, USA). During the course of testing, the samples weighing 2–3 mg were heated at a fixed heating rate of 10 °C /min from 35 to 500 °C under a nitrogen purge.

### 3.4. *In Vitro* VLBT Release from Fa-BSANPs-VLBT

The *In vitro* drug release study was carried out in phosphate buffered saline (0.15 M, pH 7.4) at 37 °C. Fa-BSANPs-VLBT suspension (0.4 mg/mL, 10 mL; with mannitol) and raw VLBT (0.4 mg/mL, 10 mL) was placed, respectively, in treated dialysis bag (MWCO3500; Sigma, St. Louis, USA), and then the dialysis bag was immersed in a 250 mL beaker containing 200 mL release buffer. The beaker was then placed on a magnetic stirring apparatus, which was in an incubator shaker at 37 °C, and the speed of the paddle was adjusted to 100 rpm. Five milliliters of sample were withdrawn at predetermined time intervals and equivalent amount of fresh release buffer was replaced. The drug content in the withdrawn samples was estimated by HPLC using conditions as described above. The accumulative release percentage was calculated using the following equations:

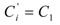
(5)

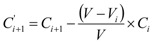
(6)

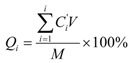
(7)

*C_i_* represents the VLBT concentration of each sample withdrawn at predetermined time intervals, *C_i_^'^* represents the increase of the VLBT concentration during each time interval, *V* represents the volume of the release buffer, *V*_0_ represents the volume of each withdrawn sample, *M* is the weight of VLBT loaded in the Fa-BSANPs-VLBT, and *Q_i_* is the accumulative release percentage at a predetermined time point. The drug release studies were carried out in triplicate for each of the samples. The drug release from optimized formulations was compared to raw VLBT, and mathematical models were applied to study the release kinetics.

### 3.5. Stability of Fa-BSANPs-VLBT

The stability of the Fa-BSANPs-VLBT study was tested with the help of DLS equipment (ZetaPALS, Brookhaven Instruments). The ultimate product along with the addition of mannitol as lyoprotectant (Fa-BSANPs-VLBT:mannitol, 1:1, w/w) were analyzed for their MPS and ZP when the pH value was 5.4 at predetermined time intervals (0, 1, 2, 4 and 8 h). The measurement conditions were determined as described above.

## 4. Conclusions

In this study, RSM was used to optimize the procedure for the preparation of VLBT-BSANPs. The results indicated that BSA concentration and the ratio of ethanol and the VLBT solution (v/v) played significant roles in determining the VLBT-BSANPs particle size, and the ratio of BSA concentration and VLBT concentration had a significant influence on DLE. The VLBT-BSANPs were obtained under optimum conditions, with a MPS of 155.4 nm and a ZP of −32.97 mV. DLE and DEE of the obtained drug were approximately up to 45.6% and 90.6%, respectively. The obtained spherically shaped Fa-BSANPs-VLBT also had a small average diameter, and their DLE and DEE were considerable. 

The nanoparticles showed a small particle size, a roughly spherical surface morphology, a high zeta potential, a high specific surface area, a sustained release property and the feature of being entirely wrapped up inside BSA. All these characterizations contributed to a good drug delivery system. Moreover, the stability experiment results showed that Fa-BSANPs-VLBT with mannitol was a suitable drug delivery system. Folate-conjugated albumin nanoparticles had a lot of advantages in the areas of cytotoxicity, anti-tumor activity *in vivo* and reduction of drug toxic action [26,34,35,36,37,38]. Therefore, Fa-BSANPs-VLBT will predictably have good effects in this respect. Furthermore, the *in vivo* properties of Fa-BSANPs-VLBT targeting drug delivery systems will be evaluated in further research. In addition, BSA will be substituted by human serum albumin in further experiments to avoid a possible immunologic response *in vivo*. 

## Abbreviations

ABC: advanced breast cancer
BSA: bovine serum albumin
BSANPs: bovine serum albumin nanoparticles
CCD: central composite design
DCC: 1,3-dicyclohexyl-carbodiimide
DEE: drug entrapment efficiency
DLE: drug loading efficiency
DLS: dynamic light scattering
DMSO: dimethyl sulfoxide
DNR: daunorubicin
DSC: differential scanning calorimetry
Fa-BSANPs: folate-conjugated bovine serum albumin nanoparticles
Fa-BSANPs-VLBT: vinorelbine tartrate loaded folate-conjugated bovine serum albumin nanoparticles
F-L-DNR: liposomes loading daunorubicin
folate-PEG-CHEMS: folate polyethylene glycol-cholesterol hemisuccinate
FTIR: Fourier transform infrared
HPLC: high performance liquid chromatography
HSA: human serum albumin
HSANPs: human serum albumin nanoparticles
L-DNR: liposomal daunorubicin
MPS: mean particle size
NHS-folate: *N*-hydroxysuccinimide ester of folate
NSCLC: non-small-cell lung cancer
RSM: response surface methodology
SEM: scanning electron microscope
TGA: thermogravimetric analyzer
VLBT-BSANPs: vinorelbine tartrate loaded bovine serum albumin nanoparticles
VLBT: vinorelbine tartrate
VLB: vinorelbine
XRD: X-ray diffraction
ZP: zeta potential
